# Potential Use of Chemoprotectants against the Toxic Effects of Cyanotoxins: A Review

**DOI:** 10.3390/toxins9060175

**Published:** 2017-05-23

**Authors:** Remedios Guzmán-Guillén, María Puerto, Daniel Gutiérrez-Praena, Ana I. Prieto, Silvia Pichardo, Ángeles Jos, Alexandre Campos, Vitor Vasconcelos, Ana M. Cameán

**Affiliations:** 1 Area of Toxicology, Faculty of Pharmacy, Universidad de Sevilla, Sevilla 41012, Spain; rguzman1@us.es (R.G.-G.); mariapuerto@us.es (M.P.); dgpraena@us.es (D.G.-P.); anaprieto@us.es (A.I.P.); spichardo@us.es (S.P.); angelesjos@us.es (A.J.); 2 Laboratory of Ecotoxicology, Genomics and Evolution, Interdisciplinary Centr of Marine and Environmental Research—CIIMAR/CIMAR, University of Porto, Terminal de Cruzeiros do Porto de Leixões, Av. General Norton de Matos s/n, Matosinhos 4450-208, Portugal; amoclclix@gmail.com (A.C.); vmvascon@fc.up.pt (V.V.); 3 Department of Biology, Faculty of Sciences, Porto University, Porto 4069-007, Portugal

**Keywords:** microcystins, cylindrospermopsin, chemoprotection, transport inhibitors, antioxidant substances

## Abstract

Cyanobacterial toxins, particularly microcystins (MCs) and cylindrospermopsin (CYN), are responsible for toxic effects in humans and wildlife. In order to counteract or prevent their toxicity, various strategies have been followed, such as the potential application of chemoprotectants. A review of the main substances evaluated for this aim, as well as the doses and their influence on cyanotoxin-induced toxicity, has been performed. A search of the literature shows that research on MCs is much more abundant than research on CYN. Among chemoprotectants, antioxidant compounds are the most extensively studied, probably because it is well known that oxidative stress is one of the toxic mechanisms common to both toxins. In this group, vitamin E seems to have the strongest protectant effect for both cyanotoxins. Transport inhibitors have also been studied in the case of MCs, as CYN cellular uptake is not yet fully elucidated. Further research is needed because systematic studies are lacking. Moreover, more realistic exposure scenarios, including cyanotoxin mixtures and the concomitant use of chemoprotectants, should be considered.

## 1. Introduction

Cyanotoxins are produced by various cyanobacteria species and are responsible for intoxications in both humans and wildlife. There is increasing interest in studies of cyanotoxins owing to their worldwide distribution and occurrence, and potential toxic blooms are more and more frequently reported. Moreover, not only can cyanotoxins be present in surface waters but also they can be accumulated in aquatic organisms and plants, some of them used as food sources [1]. Thus these toxins are of concern not only for environmental agencies but also for the European Food Safety Authority (EFSA) [2]. The most investigated group among cyanotoxins is that of microcystins (MCs), particularly MC-LR, but cylindrospermopsin (CYN) is gaining considerable importance.

The toxic effects induced by both types of toxin depend on their toxicokinetic and toxicodynamic characteristics. MCs are classified as hepatotoxins, whereas CYN is considered a cytotoxin. Toxic mechanisms reported for MCs and CYN are cyanotoxin-specific, although they also show some similarities. The resulting toxic effects are widely described in the scientific literature. These toxins have been shown to induce histopathological effects [3,4,5], genotoxicity [6,7], neurotoxicity [8,9], developmental toxicity [10,11,12], reproductive toxicity [13,14], etc. In this regard, Buratti et al. [15] recently reviewed the most relevant aspects (producing organisms, biosynthesis/genetics and occurrence, toxicological profile) of various cyanotoxins, including MCs and CYN.

In order to prevent or counteract the toxic effects induced by MCs and CYN, various chemicals have been studied in a number of experimental models, in vitro and in vivo. These chemoprotectants include inhibitors of toxin-specific transporters (rifampicin, naringin, etc.), antioxidant substances (vitamin E, vitamin C, *N*-acetylcysteine, *L*-carnitine, selenium, silymarin, lipoic acid, epigallocatechin-3-gallate, etc.), tumor necrosis factor-α antiserum, and anti-inflammatory agents, among others. Most of the reports in the scientific literature are focused on antioxidant substances that have been shown to act on oxidative stress biomarkers and histopathological damage.

The aim of this work is to synthesize, update, and compare the available information on the use of chemoprotectants to prevent/counteract the toxicity induced by MCs and CYN. These data would be useful in the decision-making process after the occurrence of cyanobacterial toxicosis and to prevent its appearance.

## 2. Microcystins

Microcystins are cyclic peptides consisting of seven amino acids, including several D-amino acids and two unusual amino acids, *N*-methyldehydroalanine (Mdha) and 3-amino-9-methoxy-2-6,8-trimethyl-10-phenyldeca-4,6-dienoic acid (Adda) [16]. More than 100 MC variants are described, MC-LR being the most studied congener, owing to its ubiquity, abundance, and toxicity [15]. The cellular uptake of these toxins [17] is mediated by the organic anion transport system (OATP).

MCs have been related to human and wildlife intoxications, with a fatal outcome in some cases. However, the true incidence is not known, although it is increasing, according to Wood [18]. Severe liver damage, intrahepatic hemorrhage, hemodynamic shock, and heart failure are the most frequent negative effects related to these cyanotoxins. Other organs affected are the kidneys, lungs, and intestines [16]. Moreover, neurotoxic effects have been reported and other organs, such as the thyroid seem to be affected [15].

With regard to the toxic mechanisms of MCs, they are mainly associated with inhibition of protein phosphatases 1 and 2A (PP1 and PP2A) and disruption of the cellular phosphorylation balance [19,20,21], affecting numerous signaling pathways. MCs can also induce oxidative stress by two primary pathways [22]. First, by glutathione (GSH) depletion, leading to oxidative damage and cell death. In fact, it is known that conjugation with GSH is the first step in the detoxication of this cyanobacterial toxin [23]. Second, increasing reactive oxygen species (ROS) production by disrupting the mitochondrial electron transport chain, leading to mitochondrial permeability transition and release of apoptotic factors, finally resulting in apoptosis [24]. Moreover, ROS can lead to increased lipid peroxidation (LPO), DNA damage, DNA-protein crosslink, and alteration of the antioxidant defense system [25,26].

With regard to the apoptosis referred to above, in addition to the mitochondrial damage already mentioned, MCs could also affect the endoplasmic reticulum (ER). MC-LR exposure causes ER distention and stress [24]. Moreover, MC-LR may produce toxic effects by causing cytoskeletal disruption. It induces the rearrangement or collapse of the three components of the cytoskeleton: microfilaments, microtubules, and intermediate filaments [24].

Finally, PP1/2A inhibition and oxidative stress exerted by MCs cause differential expression/activity of transcriptional factors and proteins involved in the pathways of cellular differentiation, proliferation, and tumor promotion [27]. MC-induced DNA damage is also involved in carcinogenicity, with MC-LR classified as a possible human carcinogen (Group 2B) by the International Agency for Research on Cancer [28]. Taking into account the scientific literature, there is a lack of studies carried out on other MC congeners different from MC-LR, such as MC-RR or MC-YR [29,30].

As has been shown, MCs use various pathways to exert their toxic effects. Therefore, the use of substances that act on the same pathways or that avoid cellular uptake of the toxin could potentially prevent or ameliorate the toxicity observed.

In view of the rapid and severe damage to the liver caused by MCs, therapy would probably have little or no value, and effective prophylaxis is critical. Despite the potential human hazards associated with MCs, there is no data available on the potential of protective strategies to treat human poisoning. Also, the development of effective chemoprotectants against these toxins in experimental models is still scarce [31,32]. Various chemically unrelated compounds have shown in vivo protection against MC toxicity in mice [33]. Among the pioneers’ works, Adams et al. [34] investigated chemicals known to affect macrophage function as potential prophylactic agents. Hermansky et al. [35,36] examined the effectiveness of several compounds, including antioxidants, enzyme inducers, calcium channel blockers, free-radical scavengers, and hepatic activity modulators, in preventing the toxicity of a lethal dose of MC-LR (100 μg/kg). Enzyme induction by phenobarbital caused partial protection, and limited protection from the lethality of MC-LR was observed in calcium channel inhibitors. This lack of effectiveness of calcium blockers might be related to a possible involvement of calcium in the toxicity of MC-LR; the toxin may cause membrane disruptions at sites that are not directly associated with calcium channels [36]. Treatment with GSH or the monoethyl ester of glutathione (GMME) i.p. 2 h before, with, and 2 h after MC-LR provided significant protection from lethality. A physical inhibition of absorption may be responsible for the protective effects; both compounds may react with MC-LR in the peritoneal cavity, preventing absorption of sufficient toxin to produce toxicity [32]. Finally, an increased hepatic GSH content of the liver may offer protection from the oxidative stress induced by MC-LR. Kaya et al. [37] reviewed neutralization of the toxicosis induced by MCs, and classified the agents as follows: (a) Bile acid transport inhibitors; (b) Antioxidants; (c) Tumor necrosis factor-α (TNF-α) antiserum; (d) Anti-inflammatory agents.

In this review, Table 1 [31,32,33,35,36,38,39,40,41,42,43,44,45,46,47,48,49,50,51,52,53,54,55,56,57,58,59,60,61,62,63,64,65] shows the various chemoprotectants found in the scientific literature that have been studied with this application, with an indication of the experimental model used (mainly in vivo models), doses or concentration of MCs or the cyanobacterial extract containing MCs which induced damage or intoxication, and the doses of chemoprotectant assayed with the most relevant effects observed against MCs. The majority of the studies have been carried out in mammals (mice), although recently more investigations have been performed on aquatic organisms. In general, analysis of the data indicates that the information available is still limited. Therefore, more laboratory experiments are needed, particularly in rats by oral route, in order to obtain a better correlation with human health.

### 2.1. Transporter Inhibitors

The prerequisite for the toxic effects of MCs appears to be their uptake into cells [66]. In this regard, the uptake of MCs into hepatic and neuronal cells has been widely characterized, partly explaining the fact that the target organs of MCs are mainly liver and brain [67]. The first insights into MC transport were observed in rat hepatocytes, where basolateral uptake of MCs was inhibited by cholate, taurocholate, and bromosulfophthalein [45,67]. These compounds are substrates of the organic anion transporting polypeptide (OATP) superfamily of membrane transporters [68,69]. OATPs are expressed in the brain (blood–brain barrier, choroid plexus) as well as in the liver [66]. However, members of the multispecific OATP family can be detected in nearly all tissues of humans, rodents, and other animals [70]. The human OATP family is composed of 11 members that share a similar structure [71]. Three of these transporters have shown the ability to transport MCs: the hepatocyte-enriched OATP 1B1 and OATP 1B3 transporters, and the ubiquitous OATP 1A2, primarily expressed at the blood–brain barrier [66,72,73]. Inhibition of these transporters has been evidenced in studies on the mechanism of entrance of MCs into cells. The possibility of using inhibitors of MC transport to avoid its toxic effects has been studied for more than 20 years.

The first experiments dealing with the use of chemoprotectants were performed in vivo to observe the prevention of death in mice exposed to MCs previously treated with cyclosporin A (CyA) [35,43]. The authors showed the importance of the time of pretreatment with this substance to avoid the lethality of MCs at doses that produce 100% death of exposed mice. However, even if the protective role of CyA could be related to the adequate prevention of MCs uptake into the hepatocyte, this was not confirmed experimentally [35]. Furthermore, Stoner et al. [43] suggested that MCs and CyA may share some unidentified membrane transport and cytosol effector mechanisms in proximal MCs target cells. Later, Runnegar et al. [45] concluded that inhibition of MCs uptake by hepatocytes was the most likely mechanism of chemoprotection for MCs in vivo toxicity for CyA as well as for other substances, such as rifampicin, trypan blue, and trypan red. They stated that, although the chemoprotectants studied were different chemically and in biological action, their protective effect must be either a common interaction with the carriers for MCs in the hepatocyte or prevention of MCs binding and uptake by the carrier. However, they were not able to gain insight into the possible transporter involved, despite having used bile acids in the experiment. In this regard, hepatocellular uptake of MC-LR was blocked by antamanide, bromosulfophthalein, rifampicin, and bile salts (cholate and taurocholate) in freshly isolated rat hepatocytes, showing that the uptake of MC-LR was through the multispecific transport system for the bile acids [67]. Similarly, Thomson and Pace [44] explained that the protective effect of several substances (rifampicin, cytochalasin, bile acids, trypan blue, and trypan red) was related to their implication in MCs uptake in the hepatocyte, highlighting the bile acid transport system. They concluded that rifampicin was the best candidate to be used as a therapeutic drug against MCs, being more effective at preventing MCs uptake at a lower concentration than bile acids in this in vitro model. This protective finding was also observed in vivo in mouse pretreated with CyA, rifampicin, and silymarin [33]. They could not give an explanation of the protective mechanism of silymarin against MC-LR lethal effects and liver damage. However, they stated that the protection observed with rifampicin and CyA could be due to the inhibited cellular uptake of MC-LR. These latter authors also evaluated the protective role of various chemoprotectants (transport inhibitors, antioxidants, osmotic agents, hepatic activity modulators, etc.) administered as either pretreatment (1, 3, and 24 h), coadministration, or post-treatment, against toxic effects of MC-LR in mice [33]. Complete protection was observed when rifampicin, CyA, and silymarin were given as pretreatment. Additionally, rifampicin and CyA exhibited complete protection when coadministered with the toxin; and only rifampicin was effective at 15 min post-treatment. Despite the higher protective action of these three transport inhibitors in comparison with the other protective agents assayed, in general, the studies concerning the use of antioxidant agents are substantially more numerous (see Table 1).

Furthermore, naringin, a grapefruit flavonoid, prevented toxic effects induced by MC-LR, such as cytoskeletal disruption and apoptotic liver cell death [51], and this protection has been linked to prevention of MCs uptake in cells [52]. Hence, naringin was able to prevent accumulation of MC-LR in hepatopancreas of the freshwater snail *Sinotaia histrica*. Bioaccumulation of MC-LR in the hepatopancreas of *S. histrica* is related to the transport mediated by OATPs. Several authors have proposed three mechanisms of MC toxicity inhibition by naringin in the cell: (1) prevention of keratin overphosphorylation induced by MCs; (2) inhibition of intestinal absorption of anionic drugs, such as glibenclamide, via inhibition of OATP-B; and (3) co-incubation with known Oatp/OATP substrates taurocholate and bromosulfophthalein [17,51,74]. Given that Xie et al. [52] proposed that OATP mediated MCs uptake into the hepatopancreas of *S. histrica*, naringin probably directly inhibited OATP, causing the decrease of MCs concentration in the hepatopancreas. The implication of OATP transporters in the protective effect of naringin against MCs was further confirmed by Takumi et al. [53]. Uptake of MC-LR was inhibited by naringin in kidney cells expressing OATP1B3. This latter mechanism was also observed in nostocyclopeptide-M1 (Ncp-M1), a cyanobacterial cyclic peptide that inhibits the hepatocyte drug transporters OATP1B3 and OATP1B1 [54]. This substance, isolated from a culture of *Nostoc* cyanobacteria strain, did not exhibit toxicity, and, similarly to naringin, Ncp-M1 has a preference for inhibition of OATP1B3. In addition, the synthetic analogue of Ncp-M1 has a specificity for the latter transporter even higher than that of the natural heptapeptide. This finding is of great importance, not only in the treatment of MCs but also in cancer, because OATP1B3 is also expressed in some aggressive cancers, where it confers apoptosis resistance.

### 2.2. Anti-Inflammatory Agents

The toxicosis caused by MCs could also be neutralized by the use of anti-inflammatory agents, because MCs stimulate the cyclooxygenase pathway of arachidonic acid metabolism in hepatocytes and macrophages [37]. Consequently, several glucocorticoids (fluocinolone, dexamethasone, and hydrocortisone) suppressed MC-induced release of free arachidonic acid and release of 6-keto prostaglandin F1α and thromboxane B2 in rat hepatocytes [75]. It was concluded that glucocorticoid therapy might be beneficial in MCs toxicosis, although these potential chemoprotectants have not been extensively studied for this purpose.

Some of the substances that act as transport inhibitors of MCs also exhibited anti-inflammatory and immunosuppressive activity, such as CyA and rifampicin. In this regard, CyA stimulated phospholipase activity and inhibited thromboxane synthase activity in macrophages without interfering with macrophage phagocytic, migratory, or monokine-releasing activity. Nevertheless, the relationship of these mechanisms of CyA and its ability to prevent MC-LR toxicity was not clarified [35]. Stoner et al. [43] suggested that CyA protection against MCs might be related to rapid, transient changes in hepatic macrophage function. Furthermore, Adams et al. [34] reported that the protection exerted by CyA against MC-LR lethality might be related to immunosuppression. In addition, the protective effects of CyA and rifampicin on the lethality of MCs were suggested to be mediated by immunomodulation, inhibition of protein synthesis, blocked cellular uptake of MCs, or a combination of these effects [36].

### 2.3. Osmotic Agents

The potential protective efficacy of certain osmotic agents, such as D-glucose, mannitol, and dihydroxy acetone (DHA), have been assayed against a lethal dose of MC-LR from cultures of *M. aeruginosa* (100 µg/kg) in mice [46]. D-glucose at 2 g/kg after 1, 3, and 24 h pretreatment increased survival time, but no protection from lethality was observed. Likewise, mannitol (2 g/kg) and DHA (50 mg/kg) had no protective effect at all the pretreatment time points. Pretreatment with mannitol 1 h prior to administration of MC-LR extended mouse survival time. Both osmotic agents, D-glucose and mannitol, increased survival time by inactivation or dilution of the toxin in the peritoneal cavity [36]. This preliminary finding is in agreement with the lack of protection provided by mannitol administered via i.v. against a lethal dose of MC-LR.

### 2.4. Antioxidants

In this section, various well-known antioxidant substances are reviewed, such as *N*-acetylcysteine (NAC), L-cysteine, vitamins (vitamin E, vitamin C), and selenium, with a special focus on their mechanisms. Moreover, other compounds less widely investigated for this application have also been considered.

#### 2.4.1. *N*-Acetylcysteine (NAC)

##### *1* *General Aspects*

*N*-acetylcysteine (NAC) is a derivative of cysteine (Cys) which presents an acetyl group in its nitrogen atom. This compound can be oxidized by many radicals, serving as a nucleophile [76]. However, the presence of the acetyl moiety reduces the reactivity of the thiol as compared with that of Cys. In comparison to Cys, NAC is not only more water soluble, and less susceptible to oxidation (and dimerization), but also more tolerable, making it a favorable source of Cys [77,78]. NAC efficiently reduces disulfide bonds in proteins, modifying their structures and affecting their ability to bond ligands, competing with larger reducing molecules, and acting as a precursor of Cys for GSH synthesis [76]. NAC participates in the GSH synthesis route providing the Cys group present in the GSH molecule [79]. Moreover, NAC enhances the activity of cytosolic enzymes involved in the GSH cycle. An example of these enzymes is glutathione reductase (GR), which increases the generation of GSH from GSSG. NAC also protects cells by direct reaction with ROS when GSH levels are depleted.

It is worth mentioning that NAC is not more effective when the concentrations used are higher. As Halliwell [80] stated, high concentrations of an antioxidant could sometimes lead to pro-oxidant activity, possibly mediated through a Fenton reaction. Puerto et al. [39] corroborated this in tilapia (*Oreochromis niloticus*) exposed to different concentrations of NAC, with the highest one (96.8 mg NAC/fish/day) presenting pro-oxidant activity.

##### *2* *Protective Effects of NAC against MCs*

All the features mentioned above make NAC a useful protective agent against substances that may cause oxidative damage, such as MCs. The efficacy of NAC to prevent or restore MC-induced toxicity has been investigated in four experimental models (Table 1): in vivo in mice [33] and fish [39,40], and in vitro in primary rat hepatocytes [38], and in Chinese hamster ovary (CHO) cells [41].

In albino female mice only partial protection from lethality with MC-LR (100 mg/kg) was observed with 1 h NAC pretreatment (15 mg/kg). With 3 and 24 h pretreatment, although 25% of the animals survived, coadministration was not effective. L-cysteine at 280 mg/kg offered no protection from lethality although it extended survival time with 24 h pretreatment [33].

In fish, the protective effects of NAC in vivo against the toxic action of MC-producing cyanobacterial cells when administered to tilapia (*O. niloticus*) before MCs exposure have been demonstrated [39,40] (Table 1). Exposure to a single dose of MC-LR (120 µg MC-LR/fish) resulted in high levels of LPO in liver and kidney, a high rate of protein oxidation, and a reduction of the protein content in the liver of the exposed fish. To face this situation, an induction of the activity of various antioxidant enzymes, such as superoxide dismutase (SOD), catalase (CAT), glutathione peroxidase (GPx), GR, and glutathione S-transferase (GST), was observed [39]. Moreover, MCs induced morphopathological changes in various organs (liver, kidney, heart, intestine, and gills) [40]. Most of these toxic effects were reverted by pretreatment with NAC for 7 days, as detailed in Table 2 [39,40,56,61,62]. Treatment with the two lower doses of NAC employed contributed to restoring oxidative parameters to control levels in liver and kidney. The protection by NAC that was observed could be due to a direct reaction of its nucleophile center with ROS, and it could act as a Cys supplier for GSH synthesis [39]. By contrast, in fish only pretreated with the highest dose of NAC, some oxidative biomarkers were increased, such as LPO, protein oxidation level, and SOD activity in comparison with the control group [39]. This is in agreement with a previous report by Sevgiler et al. [81], who demonstrated that high doses of NAC produced superoxide radicals (O_2_^−^) and H_2_O_2_. 

This NAC pretreatment in tilapia prevented, in a dose-dependent manner, histopathological changes induced by MCs in various organs. The lowest NAC dose (20.0 mg/fish/day) ameliorated but did not revert the histological alterations induced by MCs, especially glycogenic degeneration in liver, hemorrhages and hyperemia in kidney, enterocytes with altered microvilli, microhemorrhages in heart, and hyperemia and desquamations in gill lamellae, which were recovered mainly at the median dose (44.0 mg/fish/day). One explanation for NAC histopathological prevention may be its antioxidant activity. By contrast, pathological lesions were observed in all organs in fish exposed only to the highest dose of 96.8 mg NAC/fish/day [40]. Taking all these results into account, NAC can be considered a useful chemoprotectant. However, particular attention must be paid to its application dose because of its own pro-oxidant activity. Similarly, some authors found that NAC exerted its toxic effects by means of oxidative stress in fish liver at high doses such as intravenous infusion of 550 and 950 mg/kg in 48 h [82], 1 mmol/kg i.p. [83], or 400 mg/kg NAC i.p. [81].

In vitro, rat hepatocytes exposed to a microcystic cyanobacteria extract and pretreated with NAC for 6 h significantly enhanced the intracellular GSH level and reduced the cytotoxicity induced by lactate dehydrogenase (LDH) leakage, as well as cytoskeleton changes [38]. These findings support the idea that intracellular GSH plays an important role in MCs toxicity. In addition, in CHO cells, NAC (1–5 nmol/L) has been shown to protect from lesions derived from an oxidative situation and from apoptosis induced by MC-LR [41]. It maintained the activities of antioxidant enzymes, inhibited LPO, restrained ROS generation, suppressed caspase-3 activity, and attenuated apoptosis, by improving mitochondrial function and suppressing oxidative stress.

#### 2.4.2. Selenium (Se)

##### *1* *General Aspects*

Selenium (Se) is an essential micronutrient for individuals and animals, and it plays a significant physiological role in the antioxidant defense system [84]. Specifically, Se is a cofactor and structural component of GPx, which contains four Se atoms, one for each of the subunits that conform the enzyme [85]. This selenium-dependent enzyme uses GSH as a substrate in the detoxification of hydroperoxides in extra- and intracellular spaces and of lipid peroxides in cell membranes [86,87,88], and it removes the excess of potentially damaging radicals produced during oxidative stress [89,90]. Se can act both as a nutrient and as a toxicant. Se has been shown to possess potent cytotoxic effects producing ROS [91]. For this reason, it is important to consider which type of Se (organic or inorganic) should be considered as an antioxidant, because these cytotoxic effects have been related to the inorganic form [32].

##### *2* *Protective Effects of Se and Derivatives against MCs*

Gehringer et al. [59] demonstrated for the first time the protective effects of Se pretreatment in mice intoxicated with MC-LR, showing a partial recovery from histopathological alterations induced by MCs, such as hemorrhages, necrotic changes, and apoptosis, as well as a smaller decrease for glycogen in liver (Table 1). Moreover, they observed a partial recovery of alanine amino transferase (ALT) levels and a recovery of several oxidative stress biomarkers (LPO), GST activity, and a significant increase in the activity of GPx, responsible for the protective effects observed.

At present, there is only one study of aquatic organisms that investigates the potential effectiveness of Se pretreatment in tilapia fish (*Oreochromis* sp.) exposed to MCs [56] (Table 1 and Table 2). Se showed a dose-dependent protective role depending on the biomarker considered (Table 2). The liver showed the highest protective effects in comparison to the kidney. Similarly to the study carried out by Gehringer et al. [59] in mice, GPx activities increased in liver and kidney of non-intoxicated and Se-supplemented fish. To totally counteract the pathological alterations produced by MCs in kidney, heart, and intestines, the highest dose (6.0 µg Se/g diet) was required [56]. Moreover, this study demonstrated the pro-oxidant effects of Se, with increased kidney LPO values in MC-free fish; also, the highest dose of Se (6 µg/g diet) affected the basal levels of oxidative stress biomarkers (i.e., CAT activity in kidney, GSH/GSSG ratio in liver) and induced LPO and protein oxidation in non-intoxicated fish, which is related to the previously described cytotoxic effects of Se [91]. In general, these results indicated that supplementation with Se can enhance resistance to oxidative stress generated by MCs. However, the dose of Se should be carefully selected to avoid any potential negative consequence.

The Se trace element κ-selenocarrageenan (Se-Car) is a polysaccharide selenate formed by the conjugation of Se and carrageenan polysaccharide. This substance, with high biological availability, has been verified to be safe for animals and humans. Recently, its potential protective effects against MC-LR-induced hepatotoxicity in mice have been reported [32], this being the only study found in the scientific literature. Se-Car pretreatment (70 ppb Se) reduced oxidative and histopathological damage caused by this toxin, and it did not induce adverse effects *per se*. Se-Car reduced lipid and protein oxidation, and induced recovery of the antioxidant system (GST, CAT, and SOD) and serum Se level. Se-Car pretreatment significantly reduced MC-LR-induced apoptosis and necrosis. Moreover, Se-Car ameliorated mitochondrial dysfunction by inhibition of specific marker genes. Finally, this organic compound also alleviated endoplasmic reticulum (ER) stress activation, by reducing the increase of mRNA expression of eukaryotic translation initiation factor 4E binding protein 1 (eIF4EBP1) and phosphorylation of eIF2α (p-eIF2α) induced by MC-LR, suggesting that Se-Car could reduce MC-LR-induced toxicity through ER function restoration. These results suggest that Se-Car could protect mice from MC-LR intoxication.

#### 2.4.3. Vitamin E

##### *1* *General Aspects*

Vitamin E has been the most studied chemoprotective agent as a possible treatment against the toxic effects produced by MCs in various species, including mammals (mice, rats) and aquatic animals (estuarine crabs, fish). The term vitamin E covers eight related structures (α-, β-, γ-, δ-tocopherols, and α-, β-, γ-, δ-tocotrienols), all of them amphipathic with a polar chromanol ring and a phytyl chain; the differences are in the number of methyl groups and their positions in the chromanol group [92]. Among the four tocopherols and four tocotrienols found in food, only α-tocopherol meets human vitamin E requirements, and in vivo it is the most abundant and the most bioactive tocopherol, followed by β-tocopherol, γ-tocopherol, and δ-tocopherol [93,94]. Functionally, vitamin E represents a first defense against peroxidation of lipids, being the most important lipid-soluble antioxidant with chain-breaking activity present in the body tissues [95]. The antioxidant power of vitamin E encompasses the ability to donate H atoms, crossing biological membranes, and the ability of cytosolic reductants (ascorbate) to recycle tocopheroxyl radical [94]. Moreover, various authors have proposed that α-tocopherol functions by protecting long-chain polyunsaturated fatty acids, to maintain them at adequate levels for significant signaling reactions [94]. In general, vitamin E has long been considered as a cytoprotective factor with suggested roles in preventing inflammatory and degenerative processes in the liver during exposure to a range of xenobiotics, environmental pollutants, and dietary factors [96]. Additionally, vitamin E also induces protection against cancer owing to its antioxidant capacity, and against ischemic heart disease owing to its suppression of the atherosclerosis process [95].

##### *2* *Protective Effects of Vitamin E against MCs*

The first study was performed in mice, by a single i.p. injection of 170 or 340 U vitamin E 48 h before exposure to MC-LR (100 μg/kg), preventing the death of half of the animals employed [36] (Table 1). However, to determine the effects of vitamin E, most studies have focused mainly on determining antioxidant parameters. In this regard, a study carried out with mice supplemented with vitamin E for 4 weeks and treated with i.p. doses of 100 µg/kg MC-LR extract, representing 70% LD_50_, and administered every 3 days from day 8, showed a significant decrease in LPO, presumably due to its free radical-quenching ability, and a partial recovery of GST [59]. Thus, this study differed from the previous one of Hermansky et al. [36] in the sense that MC-LR was repeatedly given by i.p. injection and supplementation with vitamin E was given constantly. Vitamin E has also managed to reduce the oxidative injury to DNA (by formation of 8-hydroxydeoxyguanosine, 8-OH-dG) produced by MC-LR in mice, and this prevention depended on the concentration of antioxidant employed [63]. Similarly, in liver of rats orally administered with vitamin E and vitamin C daily for 3 days, significant reductions of ROS and LPO levels, and a recovery from the changes of ALT and aspartate amino transaminase (AST) induced by MC-LR were observed [64]. At histopathological level, this study showed that vitamins E and C produced a decrease of apoptosis and chromatin condensation in hepatocytes and prevented a reduction in membrane potential, as well as expression of Bax and Bid in MC-LR exposed mice. No more histopathological studies evaluating the efficacy of vitamin E against MC effects in mammals are available, as far as we know. In general, in mammals the studies have been performed by the i.p. route, an unrealistic exposure scenario. More studies are needed representing a real intake of the toxin (oral route).

By contrast, in aquatic animals, more organs (liver, kidney, heart, intestine, and gills) have been studied and more antioxidant parameters have been measured. Moreover, the effects were evaluated by oral exposure (immersion or diet), more relevant for the aquatic environment. Pinho et al. [60] were the first authors to evaluate the effect of vitamin E in antioxidant responses in crabs intoxicated with MC-LR. This study showed that vitamin E pretreatment of intoxicated crabs attenuated the increase in the activity of GST, compared to animals exposed to the toxin only. Moreover, CAT activity was reduced in posterior gills only, suggesting that higher concentrations of antioxidants may be needed in this tissue. Pretreatment also significantly increased nonproteic sulfhydryl groups (NP-SH), reinforcing the idea that vitamin E was present in gills, and the greater response in posterior rather than in anterior gills suggested that in the former there might be a higher production of pro-oxidants or perhaps the antioxidant concentration was starting to be limiting. This may be related to the higher activity of Na+, K+-ATPase observed in posterior gills of crabs.

Two studies were performed on fish (Tilapia, *O. niloticus*) exposed to MCs and pretreated with two different doses of vitamin E. In the first study, Prieto et al. [61] observed that LPO, protein oxidation, and various enzyme activities (SOD, CAT, GPx, GR) were ameliorated, with the highest vitamin E dose tested showing the greatest protective effects. The specific protective effects on the biomarkers assayed as a function of the dose employed, in the liver, kidney, and gills of fish, are shown in Table 2. Later, these authors also investigated different periods post-exposure to MCs (24, 48, or 72 h) in which vitamin E (as Trolox, a water-soluble analog of vitamin E) was effective to fight injuries at oxidative and histological levels in various organs (liver, kidney, intestines, gills, and heart) of tilapia. Trolox was more effective after 72 h and it helped to restore catarrhal processes with hyperemia, microvilli and mitochondrial tumefaction, necrotic and enteritis process, and myofibrolysis in the heart. However, shorter periods were necessary for the recovery of oxidative stress biomarkers. Specifically, LPO values were recovered in most organs after 24 h, and after 48 h in gills [62] (Table 2). These outcomes suggest that vitamin E could be used as a preventive measure in MC-exposed fish, which is of great interest to the aquaculture industry.

The protection offered by vitamin E suggests a potential application for treatment of liver injury and hepatotoxicity induced by MC-LR. However, some studies show that vitamin E can exhibit pro-oxidant effects [97], inversely proportional to its antioxidant activity (δ-tocopherol > γ-tocopherol > α-tocopherol) [98]. For this reason, it is necessary to define the dosages to be used to guarantee health safety. At present, the studies performed are very scarce and have many limitations, such as time of exposure, treatment dose, animal species, etc. Further studies are necessary to ensure safe and effective application of vitamin E in preventing the toxic effects produced by MCs.

#### 2.4.4. Other Antioxidants

Vitamin C is one of the most important water-soluble vitamins, also named L-ascorbic acid. L-ascorbic acid and dehydroascorbic acid are the major dietary forms of vitamin C. It takes part in several physiological functions, such as antioxidant activity, immunomodulation, and synthesis of collagen, carnitine, and neurotransmitters [99,100]. Vitamin C exhibits pro-oxidant activity that often enhances its cytotoxic effects, including cellular damage through the accumulation of hydrogen peroxide (H_2_O_2_), which leads to the arrest of tumor cell growth and the induction of tumor cell death [100].

Only one study [63] evaluated the effects of vitamin C per se to reduce the toxicity of microcystin-induced oxidative DNA damage in vivo in mice in comparison with other chemoprotectants (vitamin E and melatonin) (Table 1). Although the results suggest the efficacy of vitamin C, it was lower than that of other compounds. Moreover, another study in mice evaluated how the toxic effects of MC-LR were ameliorated by vitamin C but in concomitant exposure with vitamin E [64]. The combined exposure resulted in reduced ROS and LPO levels, and also mitigated the apoptosis induced by the mitochondrial damage induced by MC-LR by changing the expression pattern of proapoptotic proteins. Moreover, the intrahepatic hemorrhages and destruction of the liver structure induced by MC-LR were totally prevented. These two antioxidant vitamins of low molecular mass can interact with ROS directly and protect cells and tissues from oxidative stress. Vitamins C and E may act in a synergistic manner to enhance the antioxidative ability of each other [101,102,103], so it could be interesting to study the effects of combinations of various substances in order to reduce the dose of vitamin E administered.

Melatonin is an amphiphilic molecule and can easily pass through all cellular compartments, with the highest concentration recorded in mitochondria, potentially protecting them against oxidative stress [104]. It is suggested that melatonin has protective effects in the heart, liver, kidney, lung, and testis in rats and in the brain of mice [105]. This endogenous molecule is known to be a potent antioxidant [106], with anti-apoptotic [107] and anti-inflammatory effects [108]. Melatonin has been proposed to be the strongest inhibitor of oxidative DNA damage in MC-LR-intoxicated mice in comparison with vitamin C or vitamin E [63] (Table 1). In fact, it was 60- and 70-fold more effective than vitamin C or E, respectively. This might be because, firstly, melatonin is a direct free radical scavenger of the highly toxic •OH, neutralizing two •OH molecules for each melatonin molecule. Melatonin also scavenges its precursor, H_2_O_2_. It has been found that this molecule is mainly located in the nucleus, possibly contributing to the protective effect against the formation of 8-OH-dG induced by MCs in mice.

Flavonoids are a diverse family of polyphenolic compounds from plants. They are potent antioxidants, acting as free radical scavengers, metal chelators, or LPO inhibitors [109]. They also present anti-inflammatory activity, inhibition of platelet aggregation, antimicrobial and anti-tumor activities, etc. [110]. Among them, three flavonoids, quercetin, silybin, and morin, have shown different levels of protection as pretreatment in mice intoxicated with MC-LR (0.75 LD_50_) [48]. Some hepatic enzymes (ALT, AST, LDH) were high in a MC-LR treated group, and silybin and morin reversed the values to control levels at 3 days post-exposure, but no protection was observed 1 day post-exposure. All three flavonoids reversed the protein phosphatase activity to control levels at both 1 and 3 days post-exposure. Thus, the protection observed could be due to the combination of their antioxidant activity, mainly quercetin. Morin prolonged the survival of rat hepatocytes against oxidative damage, silybin helped to stabilize the plasma membrane of hepatocytes, and all three flavonoids contributed to conservation of GSH, and inhibitory action on uptake of toxin [48].

Moreover, in recent years there has been increased interest in the potential health benefits of green tea polyphenols (GTPs), primarily catechins. Among these properties, anti-blood coagulation, anti-hypertensive activity, oxidative damage repair, HIV treatment, and cancer prevention and treatment are the most prominent [111,112]. Catechins have a strong antioxidant activity, and are considered as potent scavengers of ROS [31,113]. In view of the diverse properties of GTPs, Xu et al. [31] evaluated their chemopreventive efficacy in mice pretreated with GTPs through intragastric administration at 50, 100, and 200 µg/kg/day (0–18 days) against subacute hepatotoxicity induced by 10 µg/kg/day MC-LR after 13 days. The results suggested that GTPs caused significant elevation of GSH levels and SOD activity in serum, as well as decreased hepatic LPO and serum ALT, AST, and alkaline phosphatase (ALP) activities. Moreover, the pretreatment inhibited hepatic apoptosis and reduced hepatocellular necrosis. On the basis of this evidence, the hepatoprotective effect of GTPs may be due to their antioxidant activity, mainly by the functional component of epigallocatechin-3-gallate (EGCG), and its anti-apoptotic properties [31]. This compound was also reported to possess anti-inflammatory, antioxidant, and antitumorigenic activities [114]. Several studies demonstrated that 40 µM MC-LR induced ROS generation, and vascular inflammation via NF-ĸB in umbilical vein endothelial cells (HUVECs), and directly stimulated phosphorylation of NF-ĸB [115]. These authors have subsequently demonstrated that 0–50 µM of EGCG dose-dependently attenuated MC-LR inhibition of HUVEC viability, migration, and angiogenesis [47]. Furthermore, EGCG inhibited ROS, TNF-α, and IL-6 expression in MC-LR-stimulated cells in a dose-dependent manner, increasing SOD and GSH levels, and reduced nitrite and MDA levels, suggesting that EGCG protected the cells, at least in part, in an anti-oxidative manner. Moreover, the compound suppressed MC-LR-induced NF-κB activation, and suppressed MC-LR-induced expression of ICAM-1 and VCAM-1, the most essential cell adhesion molecules, indicating that EGCG also protected in an anti-inflammatory manner. All these results suggested that EGCG might be a useful potential candidate for the prevention of vascular inflammatory processes in MC-LR-induced endothelial cells [47].

Sulforaphane (SFN), a naturally occurring isothiocyanate obtained from cruciferous vegetables (broccoli, etc.) has been reported as effective in preventing cancer [116], inflammation [117], and skin damage [118]. The preliminary results found in the literature suggested that SFN has been able to protect against MC-induced hepatotoxicity in in vitro and in vivo models. Firstly, pretreatment with 10 μM SFN for 12 h protected against MC-LR-induced damage in a variety of cultured cells, such as the human hepatocellular liver carcinoma HepG2, and the mouse embryonic fibroblast cell line NIH 3T3. SFN protected against cytotoxicity caused by MC-LR, and this response was mediated through the nuclear factor erythroid 2-related factor (Nrf2) [57]. Previously, Gan et al. [119] indicated that MC-LR treatment of HepG2 cells resulted in significant increases in the stability of the Nrf2 transcription factor in the cytoplasm and its nuclear translocation via binding to the cytosolic regulator protein Keap1. Thus, MC-LR induced up-regulation of Nrf2 in cancer cells and promoted liver cancer cell growth, and the results suggested a correlation of this factor with tumorigenesis. The protection induced by SFN against MC-LR was time-dependent and Nrf2-mediated, resulting in the encoding of proteins such as phase II enzymes: NAD(P)H quinone oxidoreductase 1 (NQO1) and heme oxygenase-1 (OH-1), associated with antioxidative and anti-inflammatory responses. Moreover, it also raised intracellular GSH level [57]. To confirm this in vitro study, Sun et al. [58] investigated in vivo the efficacy of the SFN-induced multi-mechanistic defense system against MC-induced hepatotoxicity in BALB/c mice injected with 5 μmol of SFN 12 h before injection of 40 or 50 μg/kg MC-LR group. The results confirmed the protective effect of SFN against MC-LR-induced hepatotoxicity. Moreover, the in vivo results showed that SFN significantly reduced MC-LR-induced CYP2E1 expression in the liver. In this regard, SFN is a versatile protective agent because it offers antioxidant activity, and anti-inflammatory and anti-apoptotic functions. Further preclinical and clinical studies are needed for its safe use as a potential chemoprotectant against MC-LR hepatotoxicity.

Because it is known that MC exposure can affect the expression of various classes of GST [120], chemicals that induce expression of these enzymes could be potential chemoprotectants against MC toxicity. In this regard, lipoic acid (LA) is able to control expression of some genes involved in synthesis of certain antioxidant enzymes, such as glutamate Cys ligase (GCL) and GST [121,122]. In addition, it has been reported that LA can act as a radical scavenger, direct antioxidant, chelator of metals, or regenerator of other antioxidants [123,124]. Amado et al. [49] reported, in a study on carp that received two i.p. injections (40 mg LA solution/kg) with a 24 h interval between each one, that LA may play an important role in neuro and liver protection against a toxic dose of 50 µg MC/kg. Results of GST activity in these organs suggested that LA could be a useful chemoprotectant agent, stimulating detoxification by increasing GST activity in the brain or by reversion of GST inhibition in the liver.

#### 2.4.5. Comparison of the Effectiveness of the Various Antioxidant Substances in MC-Intoxicated Fish

As described above, several antioxidant substances have been evaluated experimentally to prevent/counteract MC-mediated effects. However, the differences between these studies (with regard to the experimental model used, doses/concentrations, etc.) do not allow a systematic comparison. Consequently, only a comparison between the studies on the protective effects of NAC, Se, and vitamin E carried out in tilapias exposed to MCs under the same laboratory conditions, using the same dose and exposure route, is shown in Table 2, in order to clarify which of them might be the most effective and safe. With regard to the dose required for reversion of the negative effects of MCs, the antioxidant that needed the lowest dose to induce this effect was Se (1.8 µg/fish) [56], followed by vitamin E (0.21 mg/fish) [61,62], and NAC (44 mg/fish) [39,40]. However, NAC and Se were shown to induce some different anomalies in the redox status of fish when the highest doses were assayed. In addition, NAC was also shown to induce histopathological alterations per se when a dose of 96.8 mg/fish was administered [40], which might be related to the pro-oxidant activity described for NAC in the literature. This makes NAC the least recommended chemoprotectant among these three substances. Vitamin E has also been reported to present a pro-oxidant effect [97], although no negative effects were detected with any of the doses used by the authors [61,62], preventing alterations of the antioxidant biomarkers induced by MCs after 24 h [61]. Moreover, the recoveries of these parameters were corroborated in another study carried out by these authors, with vitamin E showing its main effect after 24 h of exposure to MCs versus 48 and 72 h [61]. This highlights the suitability of using vitamin E for prevention of the negative effects induced by MCs. Therefore, the order of preference and chemoprotectant dose proposed against MCs-intoxications in fish could be vitamin E (0.21 mg/fish) > Se (1.8 µg/fish) > NAC (44 mg/fish).

### 2.5. Diverse Mechanisms

The toxicity of MC-LR may depend on the immune system and may also be caused by cell membrane alterations. In this regard, lipopolysaccharide (LPS), a characteristic structural component of Gram-negative bacteria, can also protect against the toxicity of MC-LR in *Artemia salina* and *Daphnia* spp. [50]. In this case, pre-exposure (24 h) to 2 ng/mL of purified cyanobacterial LPS significantly raised the LC_50_ of MC-LR in invertebrates. This effect was also found when LPS and MC-LR were simultaneously administered, but appeared less effective. The decrease in susceptibility may be due to effects on detoxication enzyme pathways, with changes in GST activities. Moreover, the CYP450 system appeared to be suppressed by LPS.

## 3. Cylindrospermopsin

Cylindrospermopsin is a tricyclic alkaloid [125] whose main toxicity mechanism is the irreversible inhibition of protein synthesis [126]. It has been suggested that CYP450 metabolites may be responsible for the toxicity observed, although CYN metabolites have not been identified so far [15]. Oxidative stress is also involved in CYN toxicity, as it increases intracellular ROS production, reduces GSH [127], and induced changes in the activity of various oxidative stress biomarkers in vivo and in vitro [4,128,129]. Liver and kidney have been described as the main target organs of this toxin in vivo. However, its toxic effects have also been found in other organs, such as heart, stomach, thymus, spleen, lungs, ovaries, and brain [8,130]. Moreover, in vitro studies carried out in various mammalian cell lines have reported disruption of cell growth or cytotoxicity [129,131,132,133,134]. Furthermore, CYN has also shown genotoxic activity. Thus, CYN may exert clastogenic and aneugenic activities through induction of DNA strand breaks. Moreover, CYN upregulated some p-53 genes involved in DNA damage repair, cell cycle arrest, and apoptosis. Finally, it has been suggested that CYN could present a tumor-initiating activity in rodents [27].

As in the case of MCs, potential strategies to avoid/mitigate CYN effects are needed. Studies concerning prevention of cyanotoxin effects are scarce, almost nonexistent in the case of CYN compared to MCs, and, as far as we know, no studies have been carried out in relation to the use of transport inhibitors as chemoprotectants against CYN. This is to be expected, as the transport of CYN across the cell membrane has not been fully elucidated. Several mechanisms have been suggested, such as passive diffusion [126], facilitated transport mechanism [134], and active transport [135]. Recently, intestinal absorption of CYN was studied using the Caco-2 human cell line as a model of the intestinal epithelium [136,137]. In this regard, the first work dealing with this matter reported limited passage of the toxin through the intestinal epithelium in a time- and concentration-dependent way [136]. Furthermore, the paracellular route has been identified as an important pathway in intestinal CYN absorption, although a minor carrier-mediated transcellular transport may also be involved [137]. However, the transporters involved in cellular uptake of CYN have not been identified so far.

Moreover, work regarding protective effects against CYN has been conducted mainly on fish (*O. niloticus*) [138,139,140,141,142,143]. The other studies were carried out on aquatic invertebrates (*Artemia salina*, *Daphnia magna*, and *Daphnia galeata*), showing cyanobacterial LPS protection against subsequent exposure to CYN [50], on primary rat hepatocytes, where resveratrol appears to possess a cytoprotective effect against CYN insult [144], and on rats, in which resveratrol and L-arginine prevented CYN-induced cardiovascular, liver, and renal changes [145]. Therefore, additional systematic studies are required, especially in mammals, to establish the effective doses and best administration route for the various chemoprotectants in order to compare them and make recommendations on their potential use.

In the case of fish, the chemoprotectants assayed are antioxidants, NAC, Vitamin E, and L-carnitine. The protective effects of these compounds on CYN intoxication are summarized in Table 3 [138,139,140,141,142,143], and the most important aspects are described below.

### 3.1. Protective Effects of NAC against CYN

The effectiveness of NAC to prevent/reverse CYN-induced toxic effects has only been studied in fish. Gutiérrez-Praena et al. [138] found that CYN (pure or contained in a lyophilized cell culture) induced oxidative stress in tilapia (*O. niloticus*). Firstly, it was demonstrated that NAC pretreatment per se did not affect any of the oxidative parameters evaluated (LPO, protein oxidation, GSH/GSSG ratio, and activities of γ-glutamyl cysteine synthetase (γ-GCS), GST, GPx, and gene expression of GST and GPx) in fish that were not exposed to the toxin, indicating that this agent only ameliorates or prevents CYN-induced toxic effects, with the exception of GST and GPx gene expression. In liver and kidney, NAC improved the toxic effects induced by CYN (pure or from a cyanobacterium extract), and both doses were effective in preventing CYN-induced oxidative stress, although, in general, the highest dose induced a greater protective effect. As previously stated, NAC improved the redox situation, as it was able to recover γ-GCS activity and hence GSH levels [81], and react directly with ROS and some radicals (such as •OH, •NO_2_, CO_3_•−, HNO, and HOCl, at physiological pH). It has also been shown that NAC plays a role in gene expression of some enzymes, GST and GPx in this case [146,147]. Finally, as Gutiérrez-Praena et al. [138] pointed out, the different responses of NAC in liver and kidney could be due to kinetic differences in the two organs.

Similarly, Gutiérrez-Praena et al. [139] evaluated recovery from the histopathological changes induced by CYN in various organs of tilapia (*O. niloticus*) after pretreatment with NAC (Table 3). Both doses of NAC were able to impede the negative effects of CYN in the liver (steatosis and presence of glycogen), with the 45 mg/kg/day dose being the more effective. Similarly, with regard to the heart, only the dose of 45 mg/kg/day NAC was able to avoid the negative effects produced by CYN (myofibrolysis, edema, and hemorrhages). Kidney (glomerular atrophy, dilatation of the Bowman’s capsule, and thickening of basal membranes), GI tract, and gills were totally protected by the lower dose of 22 mg/kg/day. Globally, it can be assumed that NAC pretreatment appeared to prevent the lesions induced by CYN in a dose-dependent way. These results were also corroborated by the same authors by immunohistochemistry, a technique using CYN antibodies to detect CYN in the cells of the various organs studied. Thus, it was observable that pretreatment with NAC reduced partially (22 mg/kg/day) or totally (45 mg/kg/day) the presence of CYN in the cells of the various organs.

### 3.2. L-carnitine

#### 3.2.1. General Aspects

*L*-carnitine (β-hydroxy-γ-*N*-trimethyl ammonium-butyrate; LC) is a quaternary amine soluble in water, formed by lysine and methionine, with participation of vitamin C, among other compounds produced in animal tissues [148]. It is the biologically active stereoisomer of carnitine [149]. It plays an important function in fatty acid metabolism, by facilitating the mitochondrial membrane crossing of long-chain fatty acids for their β-oxidation, and energy metabolism, mainly in skeletal and heart muscles [150,151]. In humans, there is increasing evidence of the beneficial effects of LC supplementation in obesity, diabetes, and endocrine disorders [149,152].

The effect of LC supplementation on lipids (triglycerides, high-density lipoprotein-cholesterol, and apolipoprotein-A1) may be explained by its antioxidant potential [153]. Several works have shown that LC is also a good antioxidant [140,154,155,156]. LC seems to impede ROS formation, by chelating ferrous ions, managing to stabilize the free radicals formed on the α-carbon [157]. LC appears to exhibit its antioxidant effects also on the non-enzymatic defense system (GSH) apart from the enzymatic one, and probably protects from this damage by scavenging of free radicals, reducing LPO, maintaining GSH levels [158,159], and preventing alterations in various biomarkers of oxidative stress in fish tissues (liver and kidney) [140].

The heart is one of the most affected organs in LC or acetyl-*L*-carnitine (ALC) carrier (CAC) deficiency, as this is the main source of energy in this organ. CAC allows the fatty acyl moieties to be transported into the mitochondria for its β-oxidation by catalyzing the LC or ALC exchange. Consequently, several cardiovascular diseases arise from CAC deficiency [160], and it has been suggested that they could be ameliorated by supplementation with LC [159]. Although the mechanism(s) by which LC exerts its preventive effects in some disorders are not well defined, it seems that they are due to the recovery of normal oxidative status and myocardial energy reserves [161]. The kidney plays an important role in the metabolism of LC (it reabsorbs more than 95% of filtered LC, apart from participating in LC synthesis). LC prevented hypertension-associated renal function damage by reversion of the alterations induced in the activities, protein, and gene expressions of the antioxidant enzymes GPx, GR, and SOD [162]. It also produced up-regulation of eNOS (the enzyme responsible for nitric oxide production), and down-regulation of the renin–angiotensin system (RAS) components and NADPH oxidase subunits NOX2 and NOX4 [163]. LC seemed to improve the oxidative stress response through a specific modulation of nuclear transcription factors kappa B (NF-κB), Nrf2, and peroxisome proliferator-activated receptor alpha (PPARα). Therefore, inhibition of NADPH oxidase led to less production of superoxide anions, which may increase the expression of Nrf2 and PPARα and decrease that of NF-κB, thus enhancing the antioxidant defense systems [162]. In this regard, LC anti-inflammatory properties have also been reported by inhibition of the NF-κB pathway by suppression of ROS formation [164,165]. In general, it has been demonstrated that up-regulation of Nrf2 mRNA expression is associated with increases in antioxidant genes [166]. Thus, LC increased the activity of γ-GCS and CuZn-SOD by activation of the Nrf2—antioxidant responsive element (Nrf2-ARE) [159]. This element helps to maintain intracellular redox homeostasis in mammals [167]. Moreover, LC protects mitochondria against fatty acid stress and apoptosis induction through inhibition of mitochondrial swelling and Cyt-c release [168].

LC has been used for its beneficial effects on the growth of fish and crustaceans, as well as for feed conversion in several aquatic species [169,170,171]. It can promote growth performance by a protein-sparing effect, protect fish against high levels of xenobiotics and ammonia, and alleviate stress due to extreme changes in water temperature [151].

According to the literature reviewed, LC and its esters may be administered before, together with, or after the toxicant/injury, from minutes and hours to various weeks afterwards, depending on the experimental model. Harpaz [151] showed that dietary supplementation of fish with LC (100–4000 mg LC/kg of diet) administered for long periods (such as 120 days) managed to exert protection during exposure to xenobiotics. In tilapia fish, LC supplementation with 900 mg LC/kg diet was effective with regard to their growth and reproductive performance [172]. Other authors, however, while observing that LC treatments offered better resistance to xenobiotics, showed that it had no effect on growth or survival of tilapia [173]. However, Liu et al. [174] observed some alterations in liver and kidney of rats that may provide the caution information for the safety of supplementation with LC for long periods (8 weeks).

#### 3.2.2. Protective Effects of *L*-carnitine against CYN

Guzmán-Guillén et al. [140,143] demonstrated the usefulness of LC dietary pretreatment for the first time with 400 or 880 mg LC/kg bw (corresponding to 20 or 44 mg LC/fish/day), for 21 days, as prophylaxis for the oxidative stress and histopathological harm caused in tilapia fish (*O. niloticus*) by acute exposure to an extract of CYN from lyophilized *A. ovalisporum* cells or pure CYN (400 µg CYN/kg bw) (Table 3). LC supplementation per se did not result in any toxic effect in fish not exposed to CYN, only ameliorating or preventing the effects of the toxin. As can be seen in Table 3, both doses of LC gave total protection, fighting most of the CYN-induced alterations, with a few exceptions: no prevention of GPx activity in liver, partial prevention of increased SOD activity in kidney. Apart from this, the lack of CYN effect on some parameters in liver or kidney of intoxicated fish made it impossible to evaluate the protective effects of LC in those cases. Supplementation with LC seemed to prevent CYN-induced alterations partly because of the reasons explained above: its function in β-oxidation, counteracting ROS and metal-chelating properties, anti-ischemic action, together with its anti-inflammatory effects or its ability to increase intracellular carnitine content, improving mitochondrial oxidative phosphorylation and production of energy [175,176].

More recently, these antioxidant properties of LC also protected from the histopathological lesions caused by CYN under the same conditions as in the oxidative stress assay [143]. The lower dose of 20 mg LC/fish/day, managed to prevent completely the histological damage produced by CYN in liver, kidney, intestines, and gills, as well as morphometric damage in the proximal and distal convoluted tubules (PCT and DCT) in the kidney. However, it is noteworthy that the highest dose tested of LC (44 mg LC/fish/day) was needed to completely protect the heart from the histopathological injuries (hemorrhages) caused by CYN. A hypothesis for the heart damage prevention is that LC may have impeded the decrease found in ATP caused by CYN, by restoring the myocardium with a good content of carnitine, together with its role in fatty acid β-oxidation. This could be due to the role of LC in metabolic disorders and energy metabolism in tissues that obtain a great deal of their energy from β-oxidation, such as liver, skeletal muscle, heart, and placenta [177]. Another theory is based on the ability of LC to increase production of NO [178] and to restore myocardial energy reserves [179], which could prevent cardiac interstitial edema. The role of carnitine in the gastrointestinal tract is still under discussion, and it seems that the facility of LC in fatty acid oxidation could be mandatory to maintain the normal morphology and function of this organ in mice [177]. The mechanisms for LC protection in gills may probably be explained because it can interact with cardiolipin, protecting membranes and enhancing the impermeability of epithelial layers in the gills, by stimulation of energy metabolism.

*L*-carnitine supplementation presents some advantages compared to previous antioxidants tested for prevention of the effects produced by cyanotoxins (for details see Section 3.4). The lowest dose tested for LC (20 mg LC/fish/day) was normally enough for prevention of the histopathological injuries produced by CYN in most organs, with the exception of the heart [143]. In addition, LC managed to prevent the toxic effects of double the CYN dose (400 µg CYN/kg bw) tested in the work of Gutiérrez-Praena et al. [139] (200 µg CYN/kg bw). Moreover, carnitine is a natural compound, free from toxicity even when several grams are administered orally, as it is readily excreted. Nevertheless, despite this and the findings regarding the beneficial effects of LC against CYN insult, as well as in several disorders, care should be taken in cases of long-term LC supplementation, as there is evidence of rat liver and kidney impairment after supplementation with LC for long periods [174]. If doses are not a concern, an issue to consider would be the cost it would represent. In this regard, the application of high levels of LC may not be very cost-effective [151].

### 3.3. Protective Effects of Vitamin E against CYN

Taking into account the protection offered by Trolox (vitamin E) in MC intoxications, Guzmán-Guillén et al. [8] were pioneers in evaluating the protection offered by this chemoprotectant (700 mg vitamin E/kg bw/day, or 25 mg/fish/day, for 7 days) on the effects produced in liver and kidney of tilapia by acute exposure to CYN (Table 1 and Table 3). Changes induced in almost every oxidative stress biomarker were prevented by vitamin E supplementation in both organs studied, except in SOD activity, which was not prevented in kidney of pretreated, intoxicated fish (Table 3). Again, no changes were detectable for some parameters after CYN exposure, so the effect of the chemoprotectant could not be demonstrated in those cases. Vitamin E per se did not induce any significant changes in this enzymatic activity. The prevention of CYN-induced increase in LPO is in accordance with other antioxidants with similar doses: 22 mg NAC or 20 mg LC/fish/day [138,140]. This protection may easily be attributed to the protection of membranes from oxidative harm, as vitamin E can scavenge lipid peroxyl radicals that would create lipid peroxidation. In this way, vitamin E can prevent ROS from reacting with vital macromolecules (lipids, proteins, and DNA). Vitamin E showed its favorable effect in counteracting impairment in GST and GPx activities in liver and kidney, or in liver of exposed fish, respectively. Moreover, vitamin E per se did not alter GPx enzymatic activity [138]. In the kidney, vitamin E did not manage to restore SOD activity, though a slight enzymatic increase was observed in fish pretreated with the antioxidant, indicating that it can improve the antioxidant condition in kidney of exposed fish. Vitamin E counteracts ROS by scavenging, which could explain the increased GSH levels, similarly to LC, which increases γ-GCS and GSH in liver and kidney of intoxicated fish from 20 mg LC/fish/day [140]. Thus, supplementation with a non-enzymatic component of the antioxidant defense system, such as vitamin E, seemed to help to protect against the oxidative stress induced by CYN by two pathways: by itself and by reinforcing the enzymatic antioxidant defense system, which constitutes the first line of defense against oxidative damage. The liver is considered to be the most important organ involved in the regulation of redox metabolism; it is one of the main targets of CYN and the protective effects of vitamin E have been mainly visible in this organ. Other authors previously noted in connection with MCs and vitamin E that for better protection the chemoprotectant needed to be administered as a preventive measure before exposure to MCs, rather than as a treatment after intoxication [55,59].

With regard to prevention of CYN-induced histopathological lesions [142], the dose of 25 mg vitamin E/fish/day (similar to the lowest one employed for NAC and LC), totally prevented damage in liver, kidney, intestines, and gills. A partial but still quite evident prevention by vitamin E was detected in the heart, avoiding the myofibrolysis and reducing the presence of edema. This demonstrates a slightly higher potency of vitamin E to prevent heart injuries compared to LC, because at these doses LC was still not able to protect heart structures completely. Apart from the organs protected by NAC and LC, it was suggested for the first time that vitamin E managed to prevent the neurotoxic injuries caused by CYN in fish brains. It is important to maintain brain structures, as it functions as a center of all functions and any alteration suffered there may have fatal consequences. In this regard, vitamin E is the only chemoprotectant that could exert neuroprotective effects in CYN-exposed fish, preventing the structural lesions that might be caused by the functional ones. Morphologically, supplementation with vitamin E prevented CYN-induced increases in hepatocyte nuclear diameters, PCT and DCT diameters, and capillary diameters in kidney, as well as decreases in cardiac fiber diameters.

### 3.4. Global Comparison of Antioxidant Effectiveness in CYN-Intoxicated Fish

Pretreatment of fish with vitamin E demonstrated several advantages over other antioxidants, such as NAC or LC. For example, a NAC dose (22 mg NAC/fish) similar to that of vitamin E (25 mg vitamin E/fish) and LC (20 mg LC/fish) protected against 200 µg pure CYN/kg bw, whereas vitamin E and LC were able to exert this effect against twice the CYN dose (400 µg pure CYN/kg bw), demonstrating greater efficacy. Moreover, it appears that for recovery of protein oxidation and GST activity greater NAC doses (45 mg/fish/day) are needed. In fact, this dose was still not enough in some cases, as γ-GCS activity decreased in liver of fish [138]. Moreover, vitamin E per se did not induce any significant changes in GPx activity, whereas pretreatment with 45 mg NAC/fish/day in fish intoxicated with lyophilized cells induced GPx activity [138]. Earlier works have also documented NAC pro-oxidant effects [40,138], which remains behind vitamin E and LC for the selection of the best option. Although the pro-oxidant activity of vitamin E at high doses has been demonstrated in vivo [180], supplementation of CYN-intoxicated fish with 25 mg vitamin E/fish did not show this behavior per se [140,141,142]. Furthermore, in general, NAC offered only partial protection against histopathological changes induced by CYN (or MCs), normally needing the highest dose (45 mg NAC/fish/day) for total prevention [40,139].

No pro-oxidant effects were shown as a result of administration of LC by itself, which is an advantage. However, prevention of some oxidative stress biomarkers was better with vitamin E than with LC. As with NAC, in some cases the highest dose of 40 mg LC/fish/day was needed for total protection from some alterations, such as GST, GPx, and SOD activities in the liver, whereas the dose of vitamin E required to achieve it was lower. In any case, vitamin E showed total protection against practically all alterations found in oxidative stress biomarkers in the liver and almost all in the kidney, with the exception of SOD activity in kidney. The achievements of vitamin E regarding the prevention of morphometric alterations is similar but more complete compared to LC, as this chemoprotectant was only effective in the case of the kidney, and the toxin did not induce any of these alterations in liver and heart of fish in that experiment [143].

Therefore, taking into account the effective doses, costs, and possible side effects under the conditions assayed in the studies reviewed, the order of preference proposed for the use of chemoprotectants against CYN-intoxications in fish would be: vitamin E > LC > NAC. It is worth mentioning that this is the recommendation at the assayed doses because vitamin E has been shown to exert pro-oxidant effects at high levels of supplementation. In addition, there is a need to study prevention of damage in fish by exposure to specific combinations of cyanotoxins. Taking into account the neurotoxic effects of CYN [8] and the effectiveness of LC in ameliorating brain injuries [181], another issue for future studies could be the neuroprotection offered by LC against CYN exposure in fish, and comparison of antioxidants. For this purpose, previous studies demonstrated that using short-chain esters of LC such as ALC is recommended over free LC or its long-chain esters such as propionyl-*L*-carnitine (PLC), because of better crossing of the blood-brain barrier [181].

## 4. Conclusions

The potential efficacy of various chemoprotectants to prevent or mitigate the toxic effects induced by cyanobacterial toxins has been reviewed, particularly in relation to MC intoxications. Among the different types of compounds evaluated, antioxidant substances are the most frequently studied, with vitamin E being the one with the best profile. Available data are limited, therefore more systematic research would be necessary to define effective doses, times of exposure, and administration route for the various protective substances and cyanotoxins, depending on the species to be protected. Moreover, more realistic scenarios including exposure to cyanobacterial toxin mixtures should be considered, as well as the concomitant use of various chemoprotectants to take advantage of their potential synergistic effects. In addition, it would be of interest to elucidate the molecular mechanisms involved and the influence of these substances on other biomarkers not extensively investigated (i.e., neurotoxicity, immunotoxicity, etc.). Finally, further investigation would be required to extrapolate the published results to a human exposure scenario.

## Figures and Tables

**Table 1:** Protective effects of different chemoprotectants on microcystin (MC)-induced toxicity.

Chemoprotectant	Experimental Model Used	Doses/Concentration of MCs	Dose of Chemoprotectant	Effect of Chemoprotectant	References
*N*-acetylcysteine (NAC)	Primary rat hepatocytes	120 µg/mL from a microcystic cyanobacteria extract	NAC: 10 mM	Cells pretreated for 6 h showed reduced cytotoxicity and enhanced the intracellular GSH level.	[37]
NAC	Mouse	100 µg/kg	NAC: 15 mg/kg	NAC: 1 h pretreatment extended survival time; at 3 and 24 h pretreatment 25% of the animals survived. Coadministration was not effective.	[33]
L-cysteine (Cys)			Amifostine: 226 mg/kg	L-Cys: only at 24 h pretreatment extended survival time.	
Amifostine			L-Cys: 280 mg/kg	Amifostine: No protection though a slight increase in survival time was observed.	
Glutathione (GSH)			GSH: 2000 mg/kg	GSH: only at 3 h pretreatment protected 25% of the mice, and coadministration protected 50%.	
NAC	Tilapia (*Oreochromis niloticus*)	120 µg MC-LR/fish	20, 44, or 96.8 mg NAC/fish /day, pretreatment during 7 days	NAC reduced hepatic and renal oxidative stress induced by MCs, recovering LPO, GSH levels, and increased antioxidant enzyme activities, mainly by the lower dose. The highest dose induced alterations in SOD, GPx, and GR activities.	[39]
NAC	Tilapia (*O. niloticus*)	120 µg MC-LR/fish	20, 44, or 96.8 mg NAC/fish /day, pretreatment during 7 days	Prevention of histopathological changes in a dose-dependent way (20–44 mg/NAC/fish/day) in the liver, kidney, heart, gastrointestinal tract, and gills	[40]
NAC	CHO cells	0, 2.5, 5, and 10 µg MC-LR/mL	0, 1, and 5 nmol/L	NAC has a protective effect by increasing cell viability, decreasing ROS, elevating MMP, and reducing apoptosis index.	[41]
Cys	Mouse	10 mM MC-LR	L-Cys: 10 mM	MC-LR in Cys solution did not cause acute liver toxicity. However, MC-LR in GSH solution showed weaker acute toxicity of MC-LR than intact MC-LR.	[42]
GSH			GSH: 10 mM		
Cyclosporin A (CyA)	Mouse	100 µg/kg	CyA: 10 mg/kg	CyA: Pretreatment for 1 and 3 h showed 100% protection and extended survival time. Coadministration gave 100% protection, and post-treatment could not prevent lethality.	[33]
Naringin			Naringin: 50 mg/kg	Naringin: protected 25% of the animals after 1 h pretreatment, and at 3 and 24 h pretreatment only survival time was marginally extended.	
Rifampicin			Rifampicin: 25mg/kg	Rifampicin: pretreatment 1 h completely protected all the mice. Coadministration gave 100% protection. Post-administration 15 and 30 min 75% of the animals were protected.	
Silymarin			Silymarin: 400 mg/kg	Silymarin: protected 75% after 1 h pretreatment and 100% protection after 3 and 24 h.	
CyA	Mouse	100 μg/kg (MC-LR) i.p.	100 mg/kg	Prevented lethality when given 0.5–3 h prior to MC-LR	[35]
CyA	Mouse	1.7–1.8 × LD_50_ MC (MC-LR, -RR, -LY, -LA)	0.2 mg/mouse	Prevented 90% lethality when given up to 1 min after MC-LR administration. After 5 min no protection was observed.	[43]
Cytochalasins (Cyt)	Primary cultures of rat hepatocytes	1 μg/mL (MC-LR)	Cyt: 10 μM	Cyt and bile acids showed negative effects when administered alone to cells. Higher protective effect of Rif was observed in hepatocytes treated with lower concentrations than bile acids.	[44]
Rifampicin (Rif)			Rif: 2 μM		
Bile acids (cholic acid and deocycholate)			Bile acids: 0.1 mM		
Trypan blue (TB)			TB: 20 μM		
Trypan red (TR)			TR: 20 μM		
CyA	Isolated rat hepatocytes	320 mM MC-YM	CyA: 5 μM	Decreased accumulation of MC-YM after 30 min of exposure was observed when cells were pretreated for 1 min with chemoprotectant. The reductions observed were 37% for CyA, 26% for Rif, 30% for TB, and 66% for TR.	[45]
Rifampicin			Rif: 50 μM		
TB			TB: 20 μM		
TR			TR: 20 μM		
CyA	Mouse	100 μg/kg MC-LR	CyA: 10 mg/kg	Pretreatment with all substances provided 100% protection against lethality. However, some of the toxic effects of MC-LR (such as GSH depletion, lipid peroxidation and protein phosphatase inhibition) were observed in surviving animals up to 7 days after exposure but normalized after 14 days.	[46]
Rifampicin			Rif: 25 mg/kg		
Silymarin			Sy: 400 mg/kg		
Epigallocatechin-3-gallate	HUVECs	40 µM MC-LR	0–50 µM	EGCG reversed oxidant effects by reducing ROS and increasing SOD and GSH levels, and reduced NF-ĸB in cells. Moreover, it suppressed MC-LR-induced expression of ICAM-1 and VCAM-1, associated with inflammatory processes.	[47]
Flavonoids: quercetin, silybin, morin	Mouse	0.75 LD_50_ MC-LR: 57.5 µg/kg	Quercetin: 200 mg/kg Silybin: 400 mg/kg Morin: 400 mg/kg	The levels of the hepatic enzymes ALT, AST, and LDH were reversed to control at 3 days post-exposure. At 3 days, the PPAse activity was reversed to control values in all the flavonoid-treated groups	[48]
D-glucose	Mouse	100 µg/kg	D-glucose: 2000 mg/kg	D-glucose pretreatment increased survival time, but showed no protection from lethality. Mannitol and DHA had no protective effect at all the pretreatment time points, although mannitol extended survival time. Trolox ^®^ at 24 h pretreatment protected 25% of the animals. Only Trolox coadministered with MC-LR significantly extended survival time but it could not prevent lethality.	[33]
Mannitol			Mannitol: 2000 mg/kg		
Dihydroxy-acetone			Dihydroxy-acetone: 50 mg/kg		
Trolox			Trolox: 10 mg/kg		
Lipoic acid	Common carp (*Cyprinus carpio, Cyprinidae*)	50 µg MC/kg i.p.	40 mg/kg i.p.	Co-exposure led to an increase in GST activity in brain and reverted GST inhibition in liver.	[49]
Lipopolysaccharide	*Artemia salina* *Daphnia magna, Daphnia galeata*	*A. salina*: 2 µg/mL MC-LR *D. magna*: 1.26 µg/mL MC-LR *D. galeata*: 0.003 µg/mL MC-LR	2 ng/mL	Pre-incubation and simultaneous addition of LPS and MC-LR protected from lethal toxicity of MC-LR. The protective effect of LPS is mediated by detoxication enzyme pathways.	[50]
Naringin	Isolated rat hepatocytes	0.3–1 µM MC-LR	100 μM	Naringin prevented phosphorylation and disruption of the cytoskeleton caused by MC-LR. Moreover, dose-dependent apoptosis induced by MC-LR was suppressed by naringin.	[51]
Naringin	Freshwater snail (*Sinotaia histrica*)	13.7 mg/g D.W. MC-LR	1–10 mM	One single exposure to 1 mM naringin prevented 60% of MC-LR uptake in hepatopancreas. The uptake prevention rate was 100% when snails were continuously treated with 10 mM naringin for 8 days.	[52]
Naringin	HEK293-OATP1B3 cells	1–200 µM MC-LR	5-500 µM	Cytotoxicity of MC-LR was attenuated by naringin in a dose-dependent manner as the uptake of MC-LR into HEK293-OATP1B3 cells was inhibited by naringin.	[53]
Nostocyclopeptide-M1 (Ncp-1M)	Isolated rat hepatocytes, HEK293-OATP1B3, OATP1B1, OATP2B1	50 nM [^125^I]-MC-YR	10-20 µM	Ncp-1M inhibits the human MC-carrying transporters OATP1B1 and OATP1B3, blocking MC uptake.	[54]
Polyphenols (Green tea, GTP)	Mouse	10 μg/kg/day (MC-LR) i.p.	50, 100 and 200 μg/kg/day	GTP protected by elevating in serum antioxidant activities (GSH and SOD), reducing MDA level, inhibiting ROS, hepatocellular apoptosis, and up-regulating Bcl-2 protein expression. Multifocal liver cell degeneration and zonal coagulative necrosis were ameliorated.	[31]
Se (Sodium selenite)	Mice	75 ìg /kg MC-LR, i.p. (1 dose) sacrifice at 24 h	1.5 ìg /mouse/day, i.p. (2 weeks prior to MC-LR)	Partial recovery of histopathological alterations in liver. Increase in GST and GPx enzymatic activities.	[55]
		62 ìg /kg MC-LR, i.p.			
		(10 doses) sacrifice at 72 h	1.5 ìg /mouse/day, i.p. (6 weeks prior to MC-LR)	Recovery of body weight in liver. Partial recovery of ALT levels and glycogen (mg/g). Recovery of TBA values and GST levels. GPx activity increase.	
Se (sodium selenite)	Tilapia (*O. niloticus*)	Cyanobacterial cells containing 120 µg MC-LR/fish, 24 h	Pretreatment with 1.5, 3.0, 6.0 µg Se/g diet during 7 days	Se protection depended on the dose and the biomarker considered. The highest dose of Se could affect some oxidative stress biomarkers. The highest dose ameliorated the histopathological changes in the liver, kidney, heart, and GI tract of fish.	[56]
κ-Selenocarrageenan (Se-Car)	BALB/c mice	50 μg/kg MC-LR i.p.	90 ppb	Se-Car reduced lipid and protein peroxidation induced by MC-LR. Activities of GST and CAT were reduced and induced up-regulation of SOD. It could also abate the toxicity through ER function restoration.	[32]
Sulforaphane (SFN)	HepG2 BRL-3A NIH 3 T3	10 µM of MC-LR	10 μM	Protective response was mediated though Nrf2 pathway in vitro.	[57]
SFN	BALB/c mice	40 and 50 μg/kg MC-LR after 8 h	5 μmol	SFN activated Nrf2 pathway in vivo and the protection included activities of anti-cytochrome P450 induction, anti-oxidation, anti-inflammation, and anti-apoptosis.	[58]
Vitamin E	Mice	100 ìg /kg MC-LR, i.p.	86, 170, or 340 U i.p. 48 h prior to MC-LR	Prevention of death in 50% of the animals (up to 24 h) (with 170 or 340 U). Prevention of the serum LDH levels increase induced by MC (with 340 U).	[36]
Vitamin E (α- tocopherol acetate)	Mice	100 µg/ kg MC-LR extract, i.p. (7 doses) 100 µg/ kg MC-LR extract, i.p. (1 dose)	8.33 or 33.3 UI/mouse/day for 4 weeks 33.3 or 66.6 mg/mouse/day for 2 weeks	Partial recovery of LPO, ALT, and GST parameters compared to the control levels by reduction of LPO and ALT levels, and GST increased compared to toxin-treated control group (with 33.3 mg) in liver. Reduction of liver damage (with 33.3 and 66.6 mg). Increased time to death (with 66.6 mg)	[59]
Vitamin E	Estuarine crab (*Chasmagnathus granulatus)*	1.21 µg/kg/day MC-LR, oral injection, sacrifice on days 2 and 7	600 mg/kg bw/day (41 days)	CAT activity decreased in gills. Recovery of GST activity compared to the control levels. Increase of nonproteic sulfhydryl groups.	[60]
Vitamin E	Tilapia (*O. niloticus*)	Cyanobacterial cells containing 120 µg MC-LR/fish, commercial diet, 24 h	Pretreatment with 200 or 700 mg vitamin E/kg diet during 7 days	Vitamin E-pretreated fish showed no alteration in LPO levels, and oxidative enzymatic activities were improved. The highest dose employed gave the greater protective effects.	[61]
Vitamin E	Tilapia (*O. niloticus*)	Cyanobacterial cells containing 120 µg MC-LR/fish, with the diet. Fish were sacrificed at 24, 48, or 72 h	Pretreatment with 200 or 700 mg vit E/kg diet during 7 days	The oxidative stress biomarkers were ameliorated, and the higher protection was observed 24 h post toxin exposure. Histopathological lesions were more evidently recovered after 72 h.	[62]
Vitamin E Vitamin C Melatonin	Mice	75 ìg /kg MC-LR, i.p. (1 dose) sacrifice at 24 h	Vitamin E: 36.2 µM Vitamin C: 30.4 µM Melatonin: 0.55 µM per mouse/day, for 2 weeks	Recovery of ALT levels: Melatonin > Vitamin E > Vitamin C. Inhibition of 8-OH-dG formation in a dose-dependent manner: Melatonin > Vitamin C ≈ Vitamin E.	[63]
Vitamin E Vitamin C	Mice	60 µg/kg MC-LR, i.p., sacrifice at 12 h	200 and 250 mg/kg bw/day (3 days)	Decrease in ROS and MDA levels. Partial recovery of ALT and AST levels compared to the control levels by reduction of both parameters in liver. Protection against apoptosis and chromatin condensation produced by MC-LR. Prevented decrease in membrane potential. Recovery of Bax and Bid expression up to the control levels in liver.	[64]
Vitamin E Trolox	*Artemia franciscana* Nauplii	MC-LR (40 µg/mL) and *Microcystis aeruginosa* extract (CE, 10 mg dw/mL)	100 µg/mL antioxidant pretreatment (4 h exposure)	Both antioxidant pretreatments reduced mortality of approximately 50% at 9 h post-exposure against MC-LR, but offered little to no protection from cyanobacterial extract.	[65]

Abbreviations: ALT: Alanine amino transferase; AST: Aspartate amino transaminase; BRL-3A: Rat hepatocyte cell line; CHO: Chinese hamster ovary cells; GI: gastrointestinal; HEK293-OATP1B3 (OATP1B1, OATP2B1) cells: human embryonic kidney cells transfected with SLCO1B3 (SLCO1B1, SLCO2B1); HepG2: Human hepatocellular liver carcinoma cell line; HUVECs: Human umbilical vein endothelial cells; LDH: Lactate dehydrogenase; [^125^I]-MC-YR: MC-YR radiolabeled with ^125^I; MDA: malonyl dialdehyde; MMP: Mitochondrial membrane potential; NIH 3 T3: Mouse embryonic fibroblast cell line; Nrf2: nuclear factor erythroid 2-related factor; 8-OH-dG: 8-hydroxydeoxyguanosine; ROS: reactive oxygen species.

**Table 2:** N-acetylcysteine (NAC), Selenium (Se), and Vit E prevention of the effects induced by MCs in tilapia (*Oreochromis niloticus*), based on the alterations in some oxidative stress parameters in liver, kidney, and gills. Lipid peroxidation (LPO), protein oxidation, and catalase (CAT), superoxide dismutase (SOD), glutathione peroxidase (GPx), glutathione reductase (GR), glutathione-S-transferase (GST), and GSH/GSSG ratio, together with the histopathological changes in liver, kidney, heart, intestines, and gills of exposed fish.

Chemoprotectant	MCs Dose	Parameters Studied	Effects									Ref.
			Liver					Kidney				
NAC	120 µg MC-LR/fish, oral, 24 h	20, 44, or 96.8 mg/fish/day (7 days before intoxication)	20 mg		44 mg	96.8 mg		20 mg	44 mg		96.8 mg	[39,40]
		LPO	Total		Total	Total		Partial	Total		Alteration	
		Protein oxidation	No prevention		No prevention	No prevention		-	-		-	
		CAT	Partial		Total	Total		Total	Total		Total	
		SOD	No prevention		No prevention	No prevention		Total	Total		No prevention	
		GR	No prevention		No prevention	No prevention		Total	Total		Alteration	
		GPx	Total		No prevention	No prevention		No prevention	No prevention		Total	
		GST	Total		Total	Total		No MC effect	Alteration		Alteration	
		GSH/GSSG	Total		Total	Total		-	-		-	
		Histopathology	Liver, kidney, heart, intestines, and gills: partial recovery from toxic effects of 20 mg and total from 40 mg. Induction of toxic effect at 96.8 mg.									
Se (sodium selenite)	Cyanobacterial cells 120 µg MC-LR/fish, oral, 24 h	1.5, 3.0, and 6.0 µg Se/g diet (7 days before intoxication)	Liver					Kidney				[56]
			1.5 µg		3.0 µg	6.0 µg		1.5 µg	3.0 µg		6.0 µg	
		LPO	No prevention		Partial	Total		No prevention	Alteration		Alteration	
		Protein oxidation	Alteration		Alteration	Alteration		Alteration	Alteration		Alteration	
		CAT	Total		Partial	No prevention		No Se effect	Alteration		Alteration	
		SOD	No prevention		No prevention	Total		Total	Total		Total	
		GR	Total		Total	Total		No prevention	No prevention		No prevention	
		GPx	No prevention		Total	Total		No prevention	Partial		Total	
		GST	Partial		Partial	Total		No prevention	No prevention		No prevention	
		GSH/GSSG	Alteration		Alteration	Alteration		-	-		-	
		Histopathology	Liver: Partial (1.5 µg) and Total (3.0 and 6.0 µg) Kidney: No Se effect (1.5 µg), Partial (3.0 µg) and Total (6.0µg) Heart: Partial (1.5 and 3.0 µg) and Total (6.0 µg) Intestines: Partial (1.5 and 3.0 µg) and Total (6.0 µg)									
Vitamin E	Cyanobacterial cells 120 µg MC-LR/fish, oral, 24 h	200 or 700 mg vitamin E/kg diet (7 days before intoxication)	Liver			Kidney			Gills			[61]
			200 mg		700 mg	200 mg		700 mg	200 mg		700 mg	
		LPO	Total		Total	Partial		Total	No MC effect		No MC effect	
		Protein oxidation	No MC effect		No MC effect	-		-	-		-	
		CAT	No prevention		Total	No prevention		Total	No prevention		Total	
		SOD	Total		Total	Total		Total	Total		Total	
		GR	No MC effect		No MC effect	PT		PT	No MC effect		No MC effect	
		GPx	Alteration		Alteration	No prevention		No prevention	No prevention		No prevention	
Vitamin E (Trolox)	Cyanobacterial cells 120 µg MC-LR/fish, oral, 24, 48, or 72 h	700 mg vitamin E/kg diet (7 days before intoxication)	Liver			Kidney			Gills			[62]
			24 h	48 h	72 h	24 h	48 h	72 h	24 h	48 h	72 h	
		LPO	Partial	No prevention	No prevention	Partial	Total	Total	Partial	Partial	No prevention	
		Protein oxidation	No prevention	No MC effect	No MC effect	-	-	-	-	-	-	
		CAT	Partial	Partial	No MC effect	Alteration	No prevention	No prevention	No MC effect	Partial	No MC effect	
		SOD	Total	Partial	No prevention	No prevention	No prevention	No prevention	No MC effect	Total	Partial	
		GPx	Partial	Partial	No prevention	Partial	No prevention	No prevention	Partial	No MC effect	No MC effect	
		GR	Partial	Partial	No MC effect	Partial	No MC effect	Partial	No MC effect	No MC effect	No MC effect	
		GST	Partial	Partial	No prevention	No prevention	No MC effect	No prevention	No MC effect	No MC effect	No MC effect	
		GSH/GSSG	Total	No MC effect	No MC effect	No MC effect	No MC effect	No MC effect	No MC effect	No MC effect	No MC effect	
		Histopathology	Liver: Partial (24 h) and Total (72 h) Kidney: Partial (24 h and 72 h) Heart: Partial (24 h) and Total (72 h) Intestines: Total (24 h and 72 h) Gills: Total (24 h and 72 h)									

No prevention: no prevention of the alterations is observed; Partial: partial prevention of the alterations is observed; Total: total prevention of the alterations is observed, Alteration: the chemoprotectant itself induces alteration in intoxicated fish; No MC effect: MCs did not induce any significant alteration in this parameter.

**Table 3:** Effects of *N*-acetylcysteine, *L*-carnitine, and vitamin E supplementation on the changes induced by cylindrospermopsin (CYN) in some oxidative stress parameters in liver and kidney of tilapia fish (*O. niloticus*): lipid peroxidation (LPO), protein and DNA oxidation, activities of NADPH oxidase, glutathione-S-transferase (GST), glutathione peroxidase (GPx), superoxide dismutase (SOD), catalase (CAT), γ-glutamyl-cysteine synthetase (γ-GCS), GSH/GSSG ratio, GST and GPx gene expression, together with the histopathological and morphometric changes in liver, kidney, heart, GI tract, and gills of exposed fish.

Chemoprotectant	CYN Dose	Parameters Studied	Effects				References
NAC	200 µg/kg bw (pure and from an extract of *A. ovalisporum)*	22, 45 (mg/fish/day) 7 days	**Liver**		**Kidney**		[138,139]
			22	45	22	45	
		LPO	Total	Total	Partial	Partial	
		Protein oxidation	No CYN effect	No CYN effect	Total	Total	
		GST	Total	Total	No CYN effect	No CYN effect	
		GPx	Total	Total	No CYN effect	No CYN effect	
		γ-GCS	Partial	Partial	Total	Total	
		GSH/GSSG	Total	Total	Total	Total	
		GST gene expression	Increased	Increased	Reduced	Reduced	
		GPx gene expression	Increased	Increased	-	-	
		Histopathology	Liver and heart: partial prevention at 22 mg/fish/day, and total prevention at 45 mg/fish/day				
			Kidney, GI tract, and gills: total prevention at 22 mg/fish/day				
LC	400 µg/kg bw (pure and from an extract of *A. ovalisporum*)	20, 40 (mg/fish/day) 21 days	**Liver**		**Kidney**		[140,143]
			20	44	20	44	
		LPO	Total	Total	Total	Total	
		Protein oxidation	Total	Total	Total	Total	
		DNA oxidation	Total	Total	Total	Total	
		NADPH oxidase	Total	Total	No CYN effect	No CYN effect	
		GST	No CYN effect	No CYN effect	Total	Total	
		GPx	No prevention	No prevention	No CYN effect	No effect CYN	
		SOD	No CYN effect	No CYN effect	Partial	Partial	
		CAT	No CYN effect	No CYN effect	Total	Total	
		γ-GCS	No CYN effect	No CYN effect	Total	Total	
		GSH/GSSG	Total	Total	Total	Total	
		Histopathology	Liver, kidney, GI tract, and gills: total prevention at 20 mg/fish/day				
			Heart: no prevention at 20 mg/fish/day, and total prevention at 44 mg/fish/day				
		Morphometry	Liver and heart: No CYN effect				
			Kidney: total prevention at 20 mg/fish/day				
Vitamin E	400 µg/kg bw (pure)	25 (mg/fish/day) 7 days	**Liver**		**Kidney**		[141,142]
			25		25		
		LPO	Total		Total		
		Protein oxidation	Total		No CYN effect		
		DNA oxidation	No CYN effect		No CYN effect		
		GST	Total		Total		
		GPx	Total		No CYN effect		
		SOD	Total		No prevention		
		CAT	Total		Total		
		γ-GCS	Total		No CYN effect		
		GSH/GSSG	Total		No CYN effect		
		Histopathology	Liver, kidney, GI tract, gills, and brain: total prevention				
			Heart: partial prevention				
		Morphometry	Liver, kidney, and heart: total prevention				

No prevention: no prevention of the alterations is observed; No CYN effect: CYN did not induce any significant alteration in this parameter; Partial: partial prevention of the alterations is observed; Total: total prevention of the alterations is observed.
